# Multifunctional Roles of MicroRNAs in Schistosomiasis

**DOI:** 10.3389/fmicb.2022.925386

**Published:** 2022-06-09

**Authors:** Haoran Zhong, Yamei Jin

**Affiliations:** ^1^National Reference Laboratory for Animal Schistosomiasis, Shanghai Veterinary Research Institute, Chinese Academy of Agricultural Sciences, Shanghai, China; ^2^Key Laboratory of Animal Parasitology of Ministry of Agriculture and Rural Affairs, Shanghai Veterinary Research Institute, Chinese Academy of Agricultural Sciences, Shanghai, China

**Keywords:** schistosomiasis, MicroRNA, development, liver fibrosis, biomarker, diagnosis

## Abstract

Schistosomiasis is a parasitic disease that is caused by helminths of the genus *Schistosoma*. The dioecious schistosomes mate and lay eggs after undergoing a complex life cycle. Schistosome eggs are mostly responsible for the transmission of schistosomiasis and chronic fibrotic disease induced by egg antigens is the main cause of the high mortality rate. Currently, chemotherapy with praziquantel (PZQ) is the only effective treatment against schistosomiasis, although the potential of drug resistance remains a concern. Hence, there is an urgent demand for new and effective strategies to combat schistosomiasis, which is the second most prevalent parasitic disease after malaria. MicroRNAs (miRNAs) are small non-coding RNAs that play pivotal regulatory roles in many organisms, including the development and sexual maturation of schistosomes. Thus, miRNAs are potential targets for treatment of schistosomiasis. Moreover, miRNAs can serve as multifunctional “nano-tools” for cross-species delivery in order to regulate host-parasite interactions. In this review, the multifunctional roles of miRNAs in the growth and development of schistosomes are discussed. The various regulatory functions of host-derived and worm-derived miRNAs on the progression of schistosomiasis are also thoroughly addressed, especially the promotional and inhibitory effects on schistosome-induced liver fibrosis. Additionally, the potential of miRNAs as biomarkers for the diagnosis and treatment of schistosomiasis is considered.

## Background

Schistosomiasis is a momentous neglected tropical disease affecting countless numbers of people in the developing world that is caused by infection of three major species of trematode flukes, including *Schistosoma mansoni* (Africa, South America, and the Middle East), *Schistosoma haematobium* (Africa and the Middle East), and *Schistosoma japonicum* (Southeast Asia), and three locally distributed species, including *Schistosoma mekongi* (Mekong River area), *Schistosoma guineensis* (West and Central Africa), and *Schistosoma intercalatum* (West and Central Africa) (Colley et al., 2014). Schistosomes can live in human hosts for an average of 3–10 years and up to 40 years in extreme cases (Warren et al., 1974; Chabasse et al., 1985). Generally, the eggs produced by mated worms rather than adult worms themselves are responsible for the pathogenesis of schistosomiasis (Gryseels et al., 2006). The schistosome life cycle is relatively complex (Nation et al., 2020). Following release from freshwater snails, the cercariae pierce the skin and enter the blood vessels of the definitive host, and then transform into schistosomula (Stirewalt, 1974), which migrate through the circulation to the lungs and then to the systemic organs *via* the pulmonary veins. Finally, the male and female worms reach the liver vasculature and mate *via* the gynacophoric canal of the male (Miller and Wilson, 1980; Colley et al., 2014). The paired worms live within either the perivascular (*S. haematobium*) or mesenteric (*S. mansoni*, *S. japonicum*, *S. mekongi*, *S. guineensis*, and *S. intercalatum*) venules, which induce urogenital or intestinal/hepatic schistosomiasis, respectively (Lewis and Tucker, 2014; Nation et al., 2020).

Each mature female worm can lay hundreds or thousands of eggs per day (depending on species), of which about one-third to one-half become trapped in the host tissues, accounting for the primary pathology of schistosomiasis (Cheever et al., 1994; Zhong et al., 2022a). Egg-induced granuloma formation and tissue fibrosis due to the host immune response are the main causes of the high morbidity and mortality of schistosomiasis (Llanwarne and Helmby, 2021). Most of the remaining eggs are excreted in urine or feces. Then, miracidia hatch from the eggs in fresh water and seek a suitable snail as an intermediate host to complete and repeat the life cycle (Nation et al., 2020; Zhong et al., 2022a). Hence, schistosome eggs are also responsible for the occurrence and spread of schistosomiasis. Human activities in infected water promotes transmission of schistosomiasis (Lam et al., 2022). More than 230 million people in 78 tropical and subtropical countries are affected annually by schistosomiasis, which is the second most prevalent parasitic disease after malaria (Li et al., 2022). Infections among travelers, migrants, and immigrants are also emerging in industrialized countries, which poses significant risks worldwide (Lu et al., 2016). The sudden re-emergence of urinary schistosomiasis on the island of Corsica has aroused great concern (De Laval et al., 2014). At present, chemotherapy with praziquantel (PZQ) is mainly used to control and treat schistosomiasis (Karanja et al., 1998). However, the continued application of PZQ may promote the evolution of drug-resistant schistosomes (Fenwick et al., 2003). Hence, inhibition of schistosome growth and host-parasite interactions could contribute to the identification of novel anti-schistosomal drugs.

Small non-coding RNAs (sncRNAs), which are enriched in many cell types, are essential modulators of various processes in animal cells, including parasite infection (Bushati and Cohen, 2007; Zhu et al., 2014). MicroRNAs (miRNAs), first discovered in the nematode *Caenorhabditis elegans* in 1993, are a class of sncRNAs (about 18–25 nucleotides in length) generated from endogenous transcripts (Lee et al., 1993) that negatively regulate the expression of target genes at the post-transcriptional level by binding to the 5′-untranslated region (5′-UTR), coding sequence (CDS) and 3′-UTR of the target gene messenger RNA in order to inhibit translation or induce degradation (Bartel, 2004; Bartel, 2009). In addition to fundamental roles in diverse biological and pathological processes, miRNAs regulate the expression of genes involved in the growth, development, differentiation, proliferation, death, metabolism, and environmental responses of many organisms (Queiroz et al., 2017; Rouas et al., 2019). To date, several research groups have conducted studies to investigate and identify miRNA in different life stages of developed and undeveloped male and female worms to gain novel insights into the maturation of schistosomes and host-parasite interactions in schistosomiasis (Lin et al., 2019; Liu et al., 2019; Yu J. et al., 2019; Gaber et al., 2020; Han et al., 2020; He et al., 2020; Wang Y. et al., 2020; Abreu et al., 2021; Jiang et al., 2022). Schistosomes have numerous naturally permissive hosts (such as 46 species for *S. japonicum*, including cattle, pigs, sheep, goats, and mice) and non-adaptive hosts (such as water buffalo, *Rattus norvegicus*, *Microtus fortis*, and immunodeficient mice). Comparative studies elucidating the miRNA expression profiles of different hosts infected different developmental stages of schistosomes were also conducted (Yu X. et al., 2019; Sun et al., 2021; Tang et al., 2021; Li et al., 2022; Liu et al., 2022). The characterization of differentially expressed worm-derived or host-derived miRNAs can also be a supplementary tool for grading and diagnosis of schistosomiasis (Cai et al., 2018; Mu et al., 2019; Cai et al., 2020).

The present review focuses on recent studies evaluating the multifunctional roles of miRNAs in the development of schistosomes and host-parasite interactions of schistosomiasis. The potential applications of miRNAs as diagnostic biomarkers for schistosomiasis and as novel therapeutic targets for treatment are also discussed. These findings provide a valuable resource set of candidate targets to further explore anti-schistosomal treatment strategies.

## MicroRNAs Regulate the Development of Schistosomes

### Identification of Schistosome MicroRNAs

The complex life cycles of schistosomes reflect the need to adapt to constantly changing environmental conditions. The schistosome life cycle consists of at least seven discrete developmental stages characterized by tremendous morphological and transcriptional changes occurring within the intermediate and definitive hosts (Nation et al., 2020). The different stages of schistosome growth and development are partially regulated by various miRNAs. Xue et al. (2008) first identified *S. japonicum* miRNAs in 2008 and Simões et al. (2011) explored the miRNA profile of *S. mansoni*, which have rapidly expanded our knowledge about miRNAs. Due to the limited methods available in 2008, the stem-loop TaqMan miRNA reverse transcribed polymerase chain reaction (RT-PCR) assay was primarily used for analysis of mammalian miRNAs. Only five novel miRNAs in *S. japonicum* have been identified by constructing and screening parasite cDNA libraries of size-fractionated RNAs, which include sja-let-7, sja-miR-71, sja-bantam, sja-miR-125, and sja-miR-new1 (Xue et al., 2008). Six life stages of schistosomes were selected to investigate the expression patterns of these miRNAs, which revealed that expression of sja-miR-71 and sja-bantam peaked at the cercaria stage and then decreased significantly to the lowest levels at the schistosomulum stage (Xue et al., 2008). Sja-let-7 expression was lowest in the miracidium stage, then increased during the sporocyst and cercaria stages, and gradually decreased thereafter within the mammalian host (Xue et al., 2008). In the first report of the *S. mansoni* miRNA profile, miRNA detection with probes demonstrated that Sma-miR-71, Sma-bantam, and Sma-miR-125 were evolutionarily conserved (Simões et al., 2011). Although the expression levels of sja-miR-71 and sja-bantam decreased rapidly in *S. japonicum* schistosomula in the host lung, a strong hybridization signal was observed for both miRNAs in the same stage of *S. mansoni* (Simões et al., 2011). Two other miRNAs detected in *S. japonicum* (sja-new-1 and sja-let-7) are thought to be expressed in other life cycle stages or in undetectable amounts in *S. mansoni* (Simões et al., 2011). The first genome-wide sncRNA resource for *S. haematobium* was not published until 2018. Of all 89 miRNAs (34 novel miRNAs) identified, there were 64 miRNAs presented sex-biased transcription, suggested their potential roles in sexual differentiation and reproductive processes of *S. haematobium* (Stroehlein et al., 2018). This report further expands on previous studies of schistosome miRNAs and provides additional evidence of the conservation of miRNAs among flatworms (Stroehlein et al., 2018). Most of the schistosome miRNAs studied were identified by Solexa RNA sequencing and followed by bioinformatic analysis. Differences in the expression patterns throughout the life cycle of schistosomes suggest that these miRNAs may facilitate important roles in growth and development.

An increasing number of studies have reported the miRNA profiles of *S. japonicum*, *S. mansoni*, and *S. haematobium* at various development stages, including schistosome eggs of normally developed and underdeveloped females accumulated in various host tissues and schistosomula in the host lung and liver. Adults of both sexes have been identified by transcriptome analysis and deep-sequencing technique, which provided a broader view of parasite sncRNAs (Han et al., 2020). In addition, crucial molecules associated with miRNA biogenesis, such as Dicer, Argonaute, and Drosha, are differentially expressed at different developmental stages of schistosomes (Gomes et al., 2009; Chen et al., 2010; Luo et al., 2010; Cai et al., 2012; Cardoso et al., 2020). Hence, miRNAs appear to play important roles in schistosome development. Most studies categorize schistosome-derived miRNAs based on “the stage-specific or gender-biased” principle.

### Stage-Specific and Gender-Biased MicroRNAs in Schistosomes

Deep analysis of sncRNAs revealed that sja-miR-71b-5p, sja-miR-71, sja-miR-1, sja-miR-36-3p, and sja-miR-124-3p were highly expressed in purified eggs of *S*. *japonicum* in the host liver tissue (Cai et al., 2013a). Northern blot analysis further confirmed that members of the sja-miR-71 family were the most abundant and suggested that sja-miR-71 might perform pivotal functions during development (Cai et al., 2013a). Besides, schistosome eggs, the main causative factors of schistosomiasis, release extracellular vesicles (EVs) that contain small RNAs, including parasite-specific miRNAs, that participate in host-parasite interactions (Zhu S. et al., 2016). The functions of miRNAs in EVs released by schistosome eggs are discussed later in this review.

Male-female pairing, which is essential for sexual maturation of female worms, provides an opportunity to identify miRNAs that are crucial to the reproductive biology of female schistosomes. It has been suggested that the male schistosome ensures physical transport, corrects tissue localization, aids in feeding, and provides maturation factors for the development and egg-producing ability of the female (Loverde and Chen, 1991; Rollinson et al., 1997; Colley et al., 2014). However, the underlying mechanisms remain unknown. In, Cai et al. (2011) were the first to investigate differences in miRNA profiles of male and female *S. japonicum*. The results showed that sja-miR-1, sja-miR-7-5p, sja-miR-61, sja-miR-124-3p, sja-miR-125a, sja-miR-125b, and sja-miR-219-5p were mainly dominant in males, while sja-bantam, sja-miR-71b-5p, sja-miR-3479-5p, and sja-miR-novel-23-5p were mainly found in females, suggesting possible involvement of miRNAs in sex differentiation and maintenance. A study conducted in 2013 by Marco et al. (2013) focusing on differential expression of miRNAs in *S. mansoni* demonstrated that 10 miRNAs (sma-miR-2c, sma-miR-2d, sma-miR-2f, sma-miR-31, sma-miR-36b, sma-miR-71b, sma-miR-755, sma-miR-8437, sma-miR-8447, and sma-bantam) were more abundant in females, while expression of three miRNAs (sma-miR-1, sma-miR-61, and sma-miR-281) were higher in males. Abreu et al. (2021) recently investigated differential miRNA expression profiles in each developmental phase of *S*. *mansoni* and found that sma-miR-92a, sma-miR-250, and sma-miR-new-5-5p were enriched in adult worm pairs. In 90-day *S. haematobium* worms, 59 (66%) of 89 miRNAs, including sha-miR-71a, sha-miR-71b, sha-miR-2162, and sha-bantam, exhibited substantial female-biased transcription, while 5 (6%) others, including sha-let-7, sha-miR-1, sha-miR-7a, and sha-miR-125b, were sex-biased in males (Stroehlein et al., 2018). These results suggesting that changes in miRNA expression patterns might regulate sexual differentiation of schistosomes.

Mating of male and female *S. japonicum* occurs at as early as 15 or 16 days post-infection (dpi). At 20 dpi, the oogonium is differentiated from the spermatogonium and matured vitelline cells begin to appear in females, followed by eggshell formation at 22 dpi and laying of eggs at 24 dpi (Wang et al., 2017). In general, the paired worms are considered fully mature at 28 dpi (Colley et al., 2014; Wang et al., 2017). Therefore, 16, 20–24, and 24–26 dpi are crucial growth and developmental stages of *S. japonicum* (Wang et al., 2017; Yu J. et al., 2019). Due to the complex and prolonged life cycle of schistosomes, the dynamic influence of miRNAs on growth and development cannot be fully explained at a specific point in time. Zhu L. et al. (2016) identified 14 miRNAs enriched in males, including sja-miR-7, sja-miR-61, and sja-miR-219, and four in females, including sja-bantam, at 16, 22, and 28 days post-mating using a high-throughput sequencing method and further verified by RT-PCR. Among the four miRNAs enriched in females, sja-bantam and sja-miR-31 were predominantly localized to the ovary, as determined by the *in situ* hybridization, and sja-bantam was found to play regulatory roles in ovary development and oocyte maturation with the use of antisense RNA (Zhu L. et al., 2016).

The ability of a female worm to maintain egg production requires continuous stimulation by a mature male worm (Loverde and Chen, 1991; Kunz, 2001). A single-sex infected female worm is characterized by incomplete development with stunting and an immature reproductive system, as the vitelline glands that produce the eggshell precursors and nutrients for the egg are not fully developed (LoVerde et al., 2009; Zhong et al., 2022b). Hence, single-sex infected worms do not cause serious damage to the host because underdeveloped worms cannot produce normal eggs (Zhong et al., 2022a). Han et al. (2020) employed Solexa deep-sequencing technology to identify 36 differentially expressed miRNAs between normal and underdeveloped female worms at 25 dpi, of which 20 were up-regulated and 16 were down-regulated in mated females. Comparably, Sun et al. (2014) reported that sja-bantam was distinctly up-regulated in mated females at 23 dpi, while sja-miR-1, sja-miR-7, sja-miR-7-5p, and sja-miR-71 were up-regulated in single-sex infected females at 23 dpi. These findings imply a potential role of specific miRNAs in the sexual maturation of adult schistosomes.

The rationale for the choice of time points for comparison of miRNA profiles was mostly based on the schistosome life cycle (Yu J. et al., 2019). In order to gain a global view of miRNA profiles of schistosomes, Yu J. et al. (2019) quantified dynamic expression of miRNAs in *S*. *japonicum* at 14, 16, 18, 20, 22, 24, 26, and 28 dpi. The results showed that all 79 mature *S. japonicum* miRNAs had three similar expression clusters in male and female worms from the pairing stage, throughout development and sexual maturation, to egg production (Yu J. et al., 2019). Network analysis revealed that 15 miRNAs in cluster 1 were involved in regulating male-female and parasite-host interactions, communication, and immune responses, while 11 miRNAs in cluster 2 were associated with development and sexual maturation, and 45 miRNAs in cluster 3 might be related to metabolic regulation and synthesis of substances associated with egg laying (Yu J. et al., 2019).

The identification of related target genes would be valuable to elucidate the biological functions of miRNAs. Several groups have attempted to predict the targets of various miRNAs with the use of currently available target prediction tools, such as miRanda, TargetScanS, PicTar, RNAhybrid, and PITA (Liu et al., 2019; Du et al., 2020; Han et al., 2020; He et al., 2020; Wang L. et al., 2020). Notably, many of the putative target genes of stage-specific and gender-biased miRNAs in schistosomes are likely linked to the Wnt, transforming growth factor β (TGF-β), and chemokine signaling pathways, which reportedly are involved in the development and embryogenesis of schistosomes (Zhu L. et al., 2016; Han et al., 2020). To date, the miRBase database (version 22) (Kozomara et al., 2019) includes 79 miRNAs from mature *S. japonicum* and 225 from mature *S. mansoni*. The results of recent studies are summarized in Table 1.

**TABLE 1:** Summary of miRNAs upregulated in the different developmental stages or sexes of schistosome.

Species	Life stages or gender	miR-name	References
*S. japonicum*	Egg	sja-miR-1, sja-miR-36-3p, sja-miR-71, sja-miR-71b-5p, sja-miR-124-3p	Cai et al., 2013a
	Cercaria	sja-let-7, sja-bantam, sja-miR-1, sja-miR-2a-3p, sja-miR-2a-5p, sja-miR-2b-3p, sja-miR-2b-5p, sja-miR-7-3p, sja-miR-7-5p, sja-miR-8-3p, sja-miR-10-5p, sja-miR-31-5p, sja-miR-36-3p, sja-miR-36-5p, sja-miR-71, sja-miR-71b-3p, sja-miR-71b-5p, sja-miR-124-3p, sja-miR-125b, sja-miR-133, sja-miR-190-5p, sja-miR-219-5p, sja-miR-3479-3p, sja-miR-3479-5p, sja-miR-3485-3p, sja-miR-3487, sja-miR-3489, sja-miR-3490, sja-miR-3491, sja-miR-3492, sja-miR-3493, sja-miR-3496, sja-miR-3497, sja-miR-3499, sja-miR-3500, sja-miR-3501, sja-miR-3502, sja-miR-3503, sja-miR-3504	Xue et al., 2008; Cai et al., 2011
	Lung-stage schistosomula	sja-let-7, sja-miR-2c-3p, sja-miR-2c-5p, sja-miR-2d-3p, sja-miR-2d-5p, sja-miR-2e-3p, sja-miR-2e-5p, sja-miR-10-3p, sja-miR-61, sja-miR-124-5p, sja-miR-190-3p, sja-miR-2162-3p, sja-miR-2162-5p, sja-miR-3488, sja-miR-3494, sja-miR-3495, sja-miR-3498, sja-miR-3506	Cai et al., 2011
	16-day MM	sja-miR-2b, sja-miR-2d, sja-miR-2e, sja-miR-7, sja-miR-8, sja-miR-61, sja-miR-124, sja-miR-219, sja-miR-227, sja-miR-277b, sja-miR-2162, sja-miR-3479, sja-miR-3488	Zhu L. et al., 2016
	16-day MF	sja-miR-31	Zhu L. et al., 2016
	22-day MM	sja-miR-2b, sja-miR-2d, sja-miR-2e, sja-miR-7, sja-miR-8, sja-miR-61, sja-miR-124, sja-miR-219, sja-miR-227, sja-miR-277b, sja-miR-2162, sja-miR-3479, sja-miR-3488	Zhu L. et al., 2016
	22-day MF	sja-bantam, sja-miR-2c, sja-miR-31, sja-miR-1989	Zhu L. et al., 2016
	23-day SF	sja-miR-1, sja-miR-7, sja-miR-7-5p, sja-miR-71	Sun et al., 2014
	25-day MF	sja-bantam, sja-miR-219-3p, sja-miR-3485-5p, sja-miR-3486-5p, sja-miR-3488, sja-miR-3489, sja-miR-3490, sja-miR-3491, sja-miR-3493, sja-miR-3494, sja-miR-3495, sja-miR-3497, sja-miR-3498, sja-miR-3500, sja-miR-3501, sja-miR-3502, sja-miR-3504, sja-miR-3505, sja-miR-3506, sja-miR-3507	Han et al., 2020
	25-day SF	sja-let-7, sja-miR-1, sja-miR-2c-5p, sja-miR-7-5p, sja-miR-8-3p, sja-miR-10-5p, sja-miR-36-3p, sja-miR-36-5p, sja-miR-124-3p, sja-miR-124-5p, sja-miR-125a, sja-miR-133, sja-miR-3479-5p, sja-miR-3482-3p, sja-miR-3482-5p, sja-miR-3503	Han et al., 2020
	28-day MM	sja-miR-7, sja-miR-8, sja-miR-61, sja-miR-124, sja-miR-219, sja-miR-227, sja-miR-2162, sja-miR-3479, sja-miR-3488	Zhu L. et al., 2016
	28-day MF	sja-bantam, sja-miR-2c, sja-miR-31, sja-miR-1989	Zhu L. et al., 2016
	49-day MM	sja-let-7, sja-miR-1, sja-miR-2a-3p, sja-miR-2e-3p, sja-miR-2e-5p, sja-miR-7-5p, sja-miR-8-3p, sja-miR-10-3p, sja-miR-10-5p, sja-miR-61, sja-miR-71, sja-miR-124-3p, sja-miR-125a, sja-miR-125b, sja-miR-133, sja-miR-190-5p, sja-miR-219-3p, sja-miR-219-5p, sja-miR-227, sja-miR-307, sja-miR-2162-3p, sja-miR-3479-3p, sja-miR-3480-5p, sja-miR-3482-3p, sja-miR-3496,sja-miR-3503	Cai et al., 2011
	49-day MF	sja-bantam, sja-miR-2c-3p, sja-miR-2c-5p, sja-miR-2d-3p, sja-miR-36-3p, sja-miR-71b-5p, sja-miR-2162-5p, sja-miR-3479-5p, sja-miR-3487, sja-miR-3488, sja-miR-3489, sja-miR-3491, sja-miR-3492, sja-miR-3493, sja-miR-3494, sja-miR-3495, sja-miR-3498, sja-miR-3499, sja-miR-3505, sja-miR-3506, sja-miR-3507	Cai et al., 2011
*S. mansoni*	Lung-stage schistosomula	sma-bantam, sma-miR-71	Simões et al., 2011
	49-day MM	sma-miR-1, sma-miR-61, sma-miR-281	Marco et al., 2013
	49-day MF	sma-bantam, sma-miR-2c, sma-miR-2d, sma-miR-2f, sma-miR-31, sma-miR-36b, sma-miR-71b, sma-miR-755, sma-miR-8437, sma-miR-8447	Marco et al., 2013
	50-day adult worm pair	sma-miR-92a, sma-miR-125, sma-miR-250, sma-miR-new-5-5p	Abreu et al., 2021
*S. haematobium*	90-day MM	sha-let-7, sha-miR-1, sha-miR-7a, sha-miR-745	Stroehlein et al., 2018
	90-day MF	sha-bantam, sha-miR-71a, sha-miR-71b, sha-miR-125b, sha-miR-2162	Stroehlein et al., 2018

*SF, single-sex infected female; MF, mated female; MM, mated male.*

## MicroRNAs in the Host-Parasite Interaction

### Identification of Host-Derived MicroRNAs in Schistosomiasis

**FIGURE 1:**
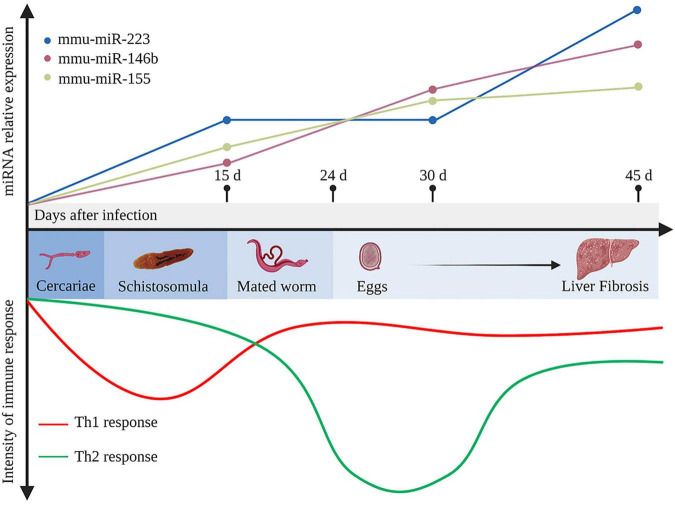
Development of the immune response and representative differentially expressed host miRNAs during *Schistosoma japonicum* infection. In the early stage of infection, the host produces a T helper type 1 (Th1) response against the migrating immature schistosomula (about 15 days after infection). After the mated worms lay eggs (about 24 days after infection), which further secrete antigens that stimulate the body to generate a strong Th2 response. Ultimately, when the disease develops into the chronic liver fibrosis, the Th2 response is diminished but still dominant. During infection, mmu-miR-223, mmu-miR-146b, and mmu-miR-155 in the liver tissues continuously elevated at 15, 30, 45 days after infection (Cai et al., 2013b). d, days. This figure was created with Biorender.com.

The enormous number of lodged eggs in the host tissues, the major pathogenic factor of schistosomiasis, triggers granuloma formation, fibrosis, and subsequent immune responses, and even cancer (Carson et al., 2018; Schwartz and Fallon, 2018). Owing to long-term coexistence within the host, schistosomes regulate the host immune responses *via* complex molecular mechanisms. The host immune response to schistosome infection is polarized during disease progression. In the early stage of infection, the host produces a T helper type 1 (Th1) response against migrating immature and mature parasites, which involves secretion of Th1-type cytokines, such as interleukin (IL) -12, interferon-γ (IFN-γ), and tumor necrosis factor-α (TNF-α). The laid eggs of paired worms (4 and 6 weeks post infection for *S. japonicum* and *S. mansoni*, respectively) act as antigens that stimulate the host to generate a strong Th2 response, with elevated production of the Th2-type cytokines IL-4, IL-5, IL-9, and IL-13, under the control of regulatory T-cells (Tregs) (Wynn et al., 2004). Ultimately, when disease progresses into the chronic regulatory phase, despite the fact that schistosomes live for years and continue to lay eggs, the Th2 response is diminished, but still dominant, due to the prolonged Treg environment in response to IL-10 and TGF-β (Pearce and MacDonald, 2002; Wynn et al., 2004; Mu et al., 2021b; Figure 1). Hyperpolarization of the Th2 immune response in the chronic phase of disease can potentially result in a lethal pathology (McManus et al., 2020). Recent observations suggest that both naïve and induced CD4^+^ T cells (Tregs) balance the host Th1/Th2 response (Layland et al., 2007; Romano et al., 2016; Ondigo et al., 2018; Wang L. et al., 2020).

Advanced liver disease induced by *S. mansoni* and *S. japonicum*, which is often irreversible without prompt medical treatment, leads to the high morbidity and mortality rates of schistosomiasis (Zhong et al., 2022a). Elucidation of the molecular mechanisms that mediate the progression of schistosomiasis has been a primary research goal for several decades. Recent studies have demonstrated that schistosome infection also alters the miRNA expression profile of the host, suggesting that the host miRNAs might also participate in regulation of the pathogenesis of schistosomiasis (Cai et al., 2013b; Han et al., 2013b; Cabantous et al., 2017).

Cai et al. (2013b) identified more than 130 differentially expressed miRNAs in the livers of BALB/c mice infected with *S. japonicum*, including mmu-miR-15b, mmu-miR-126-5p, mmu-miR-142-3p, and mmu-miR-223, which were significantly up-regulated at 30 dpi, and mmu-miR-146b and mmu-miR-155, which were up-regulated at 30 and 45 dpi. Mmu-miR-146b and mmu-miR-155 were shown to regulate the inflammatory responses *via* the nuclear factor-kappa B (NF-κB) signaling pathway to control Toll-like receptor (TLR) and cytokine signaling through a negative feedback regulation loop (Taganov et al., 2006). Hence, upregulation of mmu-miR-146b and mmu-miR-155 at 30 dpi might be associated with the recruitment and activation of B and T lymphocytes at periphery granulomas in response to stimulation by egg antigens. Expression of mmu-miR-146b and mmu-miR-155 was consistently up-regulated at 45 dpi, suggesting that dual activation of these miRNAs subtly control the degree of liver immunopathology in schistosomiasis (Cai et al., 2013b). Notably, in a mouse model of lung injury, mmu-miR-155 attenuated the Th2 response by inhibiting expression of the transcription factor *c-Maf* (Rodriguez et al., 2007). Therefore, up-regulation of mmu-miR-155 might also control the Th1/Th2 response during immunopathological progression of schistosomiasis. As another vital component, expression of mmu-miR-223 in the liver tissue is substantially up-regulated as early as 15 dpi and is reportedly the most upregulated miRNA at 45 dpi (Cai et al., 2013b). Notably, mmu-miR-223 is mainly expressed in Kupffer cells and hepatic stellate cells (HSCs), which are the most important cell types regulating liver fibrosis during schistosomiasis (He et al., 2013). In addition, miR-223 was significantly up-regulated in both cell types after *S. japonicum* infection, thus presenting a potential molecular signature of immune cell infiltration (He et al., 2013). The expression profiles of mmu-miR-146b, mmu-miR-155, and mmu-miR-223 are presented in Figure 1.

Other than the liver, the miRNA expression profiles were also identified in lung and spleen tissues of BALB/c mice in the early stage of *S. japonicum* infection (10 dpi) (Han et al., 2013b). The results of miRNA microarrays showed that that 28, 8, and 8 miRNAs were up-regulated, while 28, 3, and 5 were down-regulated, in lung, liver, and spleen tissues of infected mice, respectively (Han et al., 2013b). The lung is among the most susceptible tissues to *S*. *japonicum* in the early stage of infection (10 dpi), as reflected by the relatively large numbers of altered host miRNAs. Bioinformatics analysis demonstrated that differentially expressed miRNAs target the MAPK, insulin, TLR, and TGF-β signaling pathways, suggesting important roles of host miRNAs in the early phase of *S. japonicum* infection (Han et al., 2013b). Comparisons of plasma miRNAs between uninfected and infected (25 dpi) BALB/c mice found that 11 (30.6%) of 36 differentially expressed miRNAs were down-regulated, particularly mmu-miR-542-3p, mmu-miR-673-3p, mmu-miR-711, and mmu-miR-5112, while 25 (69.4%) were up-regulated, especially mmu-miR-874-5p (Zhu et al., 2015). Zhu et al. (2015) also found that up-regulated expression of mmu-miR-134-5p and mmu-miR-706 in the plasma was inverse correlated with levels of *Caspase-3* and *Creb*, indicating that host miRNAs may act as important mediators of the pathology of hepatic schistosomiasis.

Schistosomes have a variety of naturally permissive hosts, thus understanding the miRNA expression profiles of permissive hosts after infection can provide an important reference for the control of schistosomiasis (Liu et al., 2022). Conversely, clarification of the changes to the miRNA expression profiles of less susceptible or semi-permissive hosts can also provide constructive insights in the biology of schistosome infection (Amiri et al., 1992). Schistosomes are not highly infectious to rats with comparatively low developmental rate of the worms, decreased egg production and increased production of immature eggs (Silva-Leitão et al., 2009). Further miRNA expression profiling confirmed that rats were semi-permissive hosts of schistosomes (Han et al., 2013a). Han et al. (2013a) used miRNA microarrays to compare miRNA expression profiles of Wistar rats before and 10 days after schistosome infection and identified 16, 61, and 10 differentially expressed miRNAs in liver, spleen, and lung tissues, respectively. In liver tissues, upregulation of mmu-miR-346* was found to induce activation of the MAPK signaling pathway and rno-miR-328*, which was down-regulated in the spleen tissues of Wistar rats, was demonstrated to play important roles in activation of the Wnt, MAPK, mTOR, and neurotrophin signaling pathways (Han et al., 2013a). Water buffalo and yellow cattle are the major mammalian hosts and transmission sources of *S. japonicum* in endemic regions of China (Liu et al., 2012), and account for the majority of all infected ruminants that graze freely near bodies of water (VAN Dorssen et al., 2017). As compared with water buffalo, yellow cattles are more susceptible to schistosome infection and, therefore, the burdens of paired worms and eggs are significantly higher (Yang et al., 2012; Zhai et al., 2018). Differences in the susceptibility of yellow cattle vs. water buffalo suggest that subtle differences in gene regulation are required for the complex biological processes involved in schistosome infection. Although five miRNAs (sja-miR-2e-3p, sja-miR-7-3p, sja-miR-124-3p, sja-miR-219-5p, and sja-miR-3490) were reportedly significantly upregulated in 56-day schistosomes from water buffalo as compared to yellow cattle, the miRNA profiles of schistosome-infected bovine species are still under investigation (Yu X. et al., 2019). Collectively, these studies suggest that differences in miRNA profiles among various hosts could provide alternative strategies for the control of schistosomiasis and offer new perspectives to elucidate the underlying pathological mechanisms.

### Host MicroRNAs Mediate Schistosome-Induced Liver Fibrosis

Various stimuli can induce the differentiation of HSCs into collagen-producing myofibroblasts, which promote the progression of schistosomiasis-induced liver fibrosis (Carson et al., 2018). Recent studies have demonstrated that host miRNAs activate multiple signaling pathways that participate in the activation of HSCs during liver fibrosis (Chen et al., 2019; Sun et al., 2021; Tang et al., 2021; Xu et al., 2021). For instance, accumulating evidence suggests that miR-21 and miR-96 are involved in regulating schistosome-induced liver fibrosis through the classical TGF-β1/small mothers against decapentaplegic (SMAD) signaling pathway (He et al., 2015; Luo et al., 2018; Kong et al., 2019). Expression of miR-21 and miR-96 was inhibited in infected mice using a recombinant adeno-associated virus-8 (rAAV8) vector and further studies showed that *Smad7*, a negative regulator of the TGF-β1/SMAD signaling pathway and phosphorylation of SMAD2 and SMAD3, was the target gene of both miRNAs. Thus, miR-21 and miR-96 could activate collagen generation and stimulate liver fibrosis by targeting SMAD7 and trigger the pro-fibrotic function of the TGF-β1/SMAD signaling pathway (He et al., 2015; Kong et al., 2019). Several studies have also shown that miRNAs can target and bind to the coding regions of genes associated with the TGF-β1/SMAD signaling pathway to mediate the progression of liver fibrosis (He et al., 2020; Wang L. et al., 2020).

**FIGURE 2:**
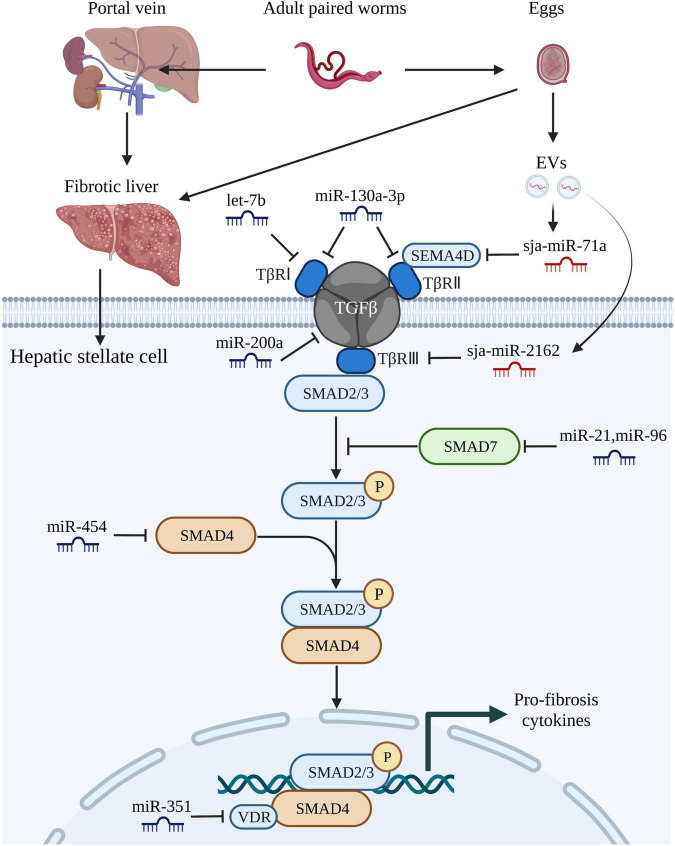
MiRNAs regulate schistosome-induced liver fibrosis through TGF-β/SMAD signaling pathway. The adult paired worms migrate to the portal vein and lay eggs. TGF-β/SMAD signaling pathway, one of the most critical signaling pathways that associated with liver fibrosis in hepatic stellate cells, can be activated by soluble egg antigen TGF-β/SMAD signaling pathway. TGF-β binds to the receptors (TβRI, TβRII, TβRIII) leading to phosphorylation of Smad-2 and Smad-3, followed by aggregation with Smad-4 and subsequently drives the expression of Smad-7 which negatively regulates TGF-β/SMAD signaling pathway. Both host-derived miRNAs (let-7b, miR-130-3p, miR-200a, miR-21, miR-96, miR-454, and miR-351) and worm-derived miRNAs secreted from egg-derived extracellular vesicles (sja-miR-71a and sja-miR-2162) exert vital roles in the progression of schistosome-induced liver fibrosis. This figure was created with Biorender.com.

Let-7b, a member of the let-7 family, plays an essential role in the development of various liver diseases (Gilles and Slack, 2018). And it was reported that let-7b diminished *S. japonicum*-induced liver fibrosis by reducing expression of TGF-β receptor 1/2 in the human HSC line LX-2, while reducing expression of collagen I, suggesting that host let-7b is a potential molecular target for the treatment of schistosome-induced liver fibrosis (Tang et al., 2017; Sun et al., 2021). In a study conducted by Liu et al. (2021), the mice injected with a lentivirus vector carrying miR-130a-3p presented alleviated granuloma-induced inflammation and collagen deposition in both liver tissue and primary HSCs. In other studies (Zhu et al., 2014; He et al., 2018b), miR-203-3p played an anti-fibrotic role by targeting SMAD3, while miR-454 exerted a similar preventive function by targeting SMAD4. Recently, miR-200a was reported to have an anti-fibrotic effect by targeting TGF-β2, although further studies are needed to determine whether miRNAs can be used to inhibit activation of the anticipated target genes (Xu et al., 2021). Liver fibrosis is a complex dynamic equilibrium, thus it is not surprising that multiple miRNAs appear to both inhibit and promote liver fibrosis. He et al. (2018a) demonstrated that miR-351 promotes schistosomiasis-related liver fibrosis by targeting the vitamin D receptor (VDR) and then they also identified a mechanism by which IFN-γ can simultaneously induce the expression of SMAD7 and VDR, which synergistically block SMAD signaling. The regulatory roles of various miRNAs associated with the TGF-β/SMAD signaling pathway in schistosome-associated liver fibrosis are summarized in Figure 2.

It is well documented that various miRNAs regulate schistosome-induced liver damage through different pathways depending on the cell type (Carson et al., 2018). As previously described, migrating schistosomes trigger a Th1 response in the host, which is characterized by increased expression of IFN-γ and subsequent differentiation of M1 macrophages. After eggs are released at 4–6 weeks post-infection, a swift transition from a Th1 to Th2 response occurs in the host. Up-regulation of miR-146a/b blocks IFN-γ-induced differentiation from M1 to M2 macrophages by targeting STAT1, suggesting a protective role against hepatic schistosomiasis (He et al., 2016). In both rat fibrotic liver tissues and LX-2 cells, high expression of miR-182 was correlated with down-regulation of the transcription factor forkhead box protein O1, which is a major downstream effector of the PI3K/AKT signaling pathway (Huang et al., 2018). Further studies have shown that increased expression of miR-182 promoted the proliferation of LX-2 cells, while inhibiting apoptosis, and stimulated the development of schistosome-induced liver fibrosis through feedback *via* the PI3K/AKT signaling pathway (Huang et al., 2018). Interestingly, another study found that miR-182 is an important mediator of the specialization and stability of Tregs during schistosome infections (Kelada et al., 2013). TLRs are crucial for the identification of invading pathogens and represent an essential bridge between innate and adaptive immunity (Akira and Takeda, 2004). Evidence suggests that miR-92a-2-5p and miR-181a suppress schistosome-induced liver fibrosis both *in vitro* and *in vivo* by targeting TLR2 and TLR4, respectively (Zhao et al., 2019; Tang et al., 2021). Egg antigen P40 of *S. japonicum* (SjP40), which is the main soluble egg antigen of *S. japonicum*, can suppress activation and proliferation of HSCs *via* TGF-β1 (Sun et al., 2015). Moreover, in two other studies, recombinant SjP40 treatment can make LX-2 cells activated by blocking the function of peroxisome proliferator-activated receptor gamma (PPARγ) through the expression of miR-27b, PPARγ is thought to inhibit fibrosis by maintaining HSCs in a more quiescent phenotype, while miR-155 inhibited activation of HSCs *via* FOXO3a (Zhu et al., 2018a,b). The results of these studies indicate indirect involvement of miRNAs in schistosome-induced liver fibrosis.

In summary, numerous studies have demonstrated that certain host-derived miRNAs play important roles in the pathogenesis of schistosomiasis and that the use of antisense techniques to block specific miRNAs and methods to improve the expression of certain miRNAs will likely provide a theoretical basis for reducing the pathological consequences of schistosomiasis.

### MicroRNAs Mediate Cross-Species Host-Parasite Interaction Through Extracellular Vesicles

EVs are released from almost all cell types, including parasitic cells, but through different mechanisms (Hansen et al., 2019; Liu et al., 2019; Wang L. et al., 2020). The three main subtypes of EVs (50–1,000 nm in diameter and can even up to 10 μm) are microvesicles, exosomes, and apoptotic bodies (van Niel et al., 2018). Notably, among the diverse active vectors conveyed by EVs, miRNAs are regarded as regulators of various biological functions. Some studies have reported that schistosome-derived EVs mediate host-parasite interactions through miRNAs. Liu et al. (2019), who first reported EVs from adult *S. japonicum* worms and determined the miRNA profiles, found that sja-miR-125b (most enriched) and sja-bantam (helminth-specific miRNA) in SjEVs stimulated the proliferation of monocytes in infected mice and elevated expression of TNF-α by targeting the genes coding for protein S1, family with sequence similarity 212 member B, and CXADR-like membrane protein. In this study, treatment with clodronate liposomes of the infected mice resulted in remarkable reduction in worm burden, egg production, and elevated expression of TNF-α, this demonstrated the pivotal role of SjEV miRNA in mediating host-pathogen interactions. Other than worms, EVs have also been identified in schistosome eggs (Zhu S. et al., 2016). Based on the miRNA profiles of schistosome eggs, the same research group also detected sja-miR-2162 and sja-miR-1 in the HSCs of infected hosts, suggesting that EVs could deliver parasite miRNAs into HSCs (He et al., 2020; Wang Y. et al., 2020). Delivery of sja-miR-2162 and sja-miR-1 *via* the rAAV8 vector could further promote activation of HSCs and induction of liver fibrosis by targeting negative modulators of HSC activation, while inhibiting expression of the miRNAs was found to attenuate the severity of liver fibrosis both *in vivo* and *in vitro* (Chu et al., 2011; He et al., 2020; Wang Y. et al., 2020).

As previously described, miRNAs both positively and negatively regulate the progression of liver fibrosis. Previous studies identified sja-miR-71a as the most enriched miRNA in *S. japonicum* egg-derived EVs and a negative modulator of liver fibrogenesis that targets *Sema4d* and negatively regulates functions of the TGF-β1/SMAD and IL-13/signal transducer and activator of transcription 6 signaling pathways during infection (Du et al., 2016; Xu et al., 2016; Wang L. et al., 2020). Besides, the proportions of Th2 and Th17 cells were decreased, while the number of Tregs was increased in mice treated with the rAAV9 vector expressing sja-miR-71a (Wang L. et al., 2020).

Recent studies have found that the parasitic miRNAs sja-miR-7-5p, sja-miR-61, and sja-miR-3096 have significant negative effects on the growth of hepatoma cell *in vitro* and *in vivo*, but no effect in normal non-cancerous cells (Hu et al., 2019; Lin et al., 2019; Hu et al., 2021). Although the current study does not confirm the involvement of SjEVs during liver cancer, in-depth studies of cross-species miRNA regulation may help to discover new strategies for the prevention and treatment of parasitic diseases, including schistosomiasis. Recent studies of host- and schistosome-derived miRNAs in the pathogenicity of schistosomiasis are summarized in Table 2.

**TABLE 2:** Summary of miRNAs involved in the pathogenesis of schistosomiasis.

MiR-name	Target genes	Pathways	Function	References
Let-7b	TβRI	TGF-β/SMAD signaling pathway	Anti-fibrosis	Tang et al., 2017
MiR-21	SMAD7	TGF-β/SMAD and IL-13/STAT6 signaling pathways	Pro-fibrosis	Kong et al., 2019
MiR-27b	PPARγ	TGF-β signaling pathway	Pro-fibrosis	Zhu et al., 2018a
MiR-92a-2-5p	TLR2	Unstudied	Anti-fibrosis	Zhao et al., 2019
MiR-96	SMAD7	TGF-β/SMAD signaling pathway	Pro-fibrosis	Luo et al., 2018
MiR-130a-3p	MAPK1, TβRI, TβRII	TGF-β and MAPK signaling pathways	Anti-fibrosis	Liu et al., 2021
MiR-146a/b	STAT1	IFN-γ signaling pathway	Macrophage transformation	He et al., 2016
MiR-155	FOXO3a	ERK1 signaling pathway	Pro-fibrosis	Zhu et al., 2018b
MiR-181a	TLR4	TLR4 receptor signaling pathway	Anti-fibrosis	Tang et al., 2021
MiR-182	FOXO1	PI3K/AKT signaling pathway	Pro-fibrosis	Huang et al., 2018
MiR-200a	TGF-β2	TGF-β signaling pathway	Anti-fibrosis	Xu et al., 2021
MiR-203-3p	SMAD3	SMAD signaling pathway	Anti-fibrosis	He et al., 2018b
MiR-351	VDR	SMAD and IFN-γ signaling pathways	Pro-fibrosis	He et al., 2018a
MiR-454	SMAD4	SMAD signaling pathway	Anti-fibrosis	Zhu et al., 2014
Sja-miR-1	SFRP1	Wnt/β-catenin pathway	Pro-fibrosis	Wang Y. et al., 2020
Sja-miR-71a	SEMA4D, FZD4	TGF-β/SMAD and IL-13/STAT6 signaling pathways	Anti-fibrosis	Wang L. et al., 2020; Jiang et al., 2022
Sja-miR-125b, Sja-bantam	PROS1, FAM212B, CLMP	Unstudied	Macrophage proliferation	Liu et al., 2019
Sja-miR-2162	TβRIII	TGF-β/SMAD signaling pathway	Pro-inflammatory, Pro-fibrosis	He et al., 2020
Sja-miR-3096	PIK3C2A	Unstudied	Tumor suppressor	Lin et al., 2019

## MicroRNAs for Grading Schistosome-Induced Liver Fibrosis

Unless treated in a timely manner, schistosome-induced liver fibrosis is irreversible. Hence, it is urgent to develop novel methods for early diagnosis and monitoring of schistosomiasis. Studies have shown that circulating and EV-bound miRNAs are potential diagnostic and prognostic markers of schistosomiasis. For example, the level of serum miR-223 was positively correlated with the course of schistosomiasis in mice, while treatment with PZQ reduced miR-223 expression to normal levels, indicating that miR-223 is a potential diagnostic biomarker of schistosomiasis (He et al., 2013). Host circulating let-7a-5p, let-7d-5p, miR-146a-5p, and miR-150-5p were identified in infected C57BL/6 and BALB/c mice, and miR-150-5p demonstrated the best potential for grading liver fibrosis in schistosomiasis (Cai et al., 2018). Besides, the serum level of exosomal miR-146a-5p was negatively correlated with the extent of liver fibrosis in patients, indicating that miR-146a-5p is also a potential novel marker to assess the severity of schistosomiasis (Cai et al., 2018). In another study, exosomal miR-103a-3p and miR-425-5p were determined as the most stable reference genes (RGs) in a C57BL/6 mouse model of schistosomiasis and human patients, respectively (Cai et al., 2020). The study demonstrated that the serum exosomal miR-146a-5p level was able to discriminate the schistosomiasis japonica patients with mild from severe fibrosis liver fibrosis (Cai et al., 2020).

Detection of parasite-specific miRNAs in exosomes could improve diagnostic accuracy. For returning travelers infected with schistosomiasis due to infection with *S. mansoni*, *S. haematobium*, and *S. mekongi*, the diagnostic sensitivity and specificity of parasite-specific miRNAs, including Bantam, miR-2c-3p, and miR-3488, in human serum EVs are much higher than other diagnostic methods, as the sensitivity of Bantam and miR-2c-3p combined was 91% (Meningher et al., 2017). RT-PCR analysis conducted by Mu et al. (2019) demonstrated that parasite-derived miRNAs (sja-miR-2b-5p and sja-miR-2c-5p) could detect infected patients with low infectious intensity and moderate sensitivity and specificity of 66/68% and 55/80%, respectively. Notably, in this study they also presented that the sensitivity and specificity of these two parasite miRNAs combined were 77.4 and 60.0%, respectively.

Current techniques for extraction and purification of EVs must be improved because the number of exosomes in serum is limited. Timely and rapid extraction of exosomes and efficient detection of substances contained in exosomes remains difficult (Mu et al., 2021a). Focusing on the schistosome-derived miRNAs to diagnose schistosomiasis is a promising path, but more research is needed before clinical application.

## Conclusion

Emerging studies have indicated that miRNAs play important roles in the development of schistosomes and host-parasite interactions. Due to the complex life-cycle of schistosomes (at least seven life stages) and the variety of host species (over 40 permissive hosts for *S. japonicum*), further studies are needed to clarify the contribution of parasite-derived miRNAs in schistosome growth. Mortality associated with schistosome-induced liver disease is relatively high and recent studies suggest that miRNAs of either the host or schistosome are involved in fatal host-parasite interactions. Current studies of the miRNA expression profiles of the host liver and serum offer important insights into the pathogenesis of schistosomiasis. Interestingly, some differentially expressed miRNAs identified in these studies regulate schistosome-induced liver disease positively or negatively, suggesting that miRNAs are potential diagnostic and prognostic markers of schistosomiasis. However, miRNA-based therapy is still in the animal experimental stage and in-depth investigations are still needed. Screening of worm-specific miRNAs from schistosome-derived EVs may also be a good approach for choosing potential biomarkers of schistosomiasis. Although still in the infancy period, recent studies have focused on cross-species miRNAs. However, there still exist significant discrepancies in this concept, especially from the perspective of the basic biology of cross-species miRNAs. The extraction, purification, and substance identification of EVs still require optimization and improvement. In all, investigations of schistosomiasis-related miRNAs should be pursued to provide a theoretical basis for the growth and development of schistosomes as well as the diagnosis and treatment of schistosomiasis.

## Author Contributions

YJ and HZ: conceptualization, validation, investigation, resources, and writing – review and editing. HZ: writing – original draft preparation and visualization. YJ: supervision, project administration, and funding acquisition. Both authors have read and agreed to the published version of the manuscript.

## Conflict of Interest

The authors declare that the research was conducted in the absence of any commercial or financial relationships that could be construed as a potential conflict of interest.

## Publisher’s Note

All claims expressed in this article are solely those of the authors and do not necessarily represent those of their affiliated organizations, or those of the publisher, the editors and the reviewers. Any product that may be evaluated in this article, or claim that may be made by its manufacturer, is not guaranteed or endorsed by the publisher.

## Abbreviations

EV: extracellular vesicles
Fox: forkhead box
HSC: hepatic stellate cells
IFN- γ: interferon- γ
IL: interleukin
LV: lentivirus vector
mRNA: messenger RNA
miRNA: microRNA
NF- κ B: nuclear factor- kappa B
PPAR γ: peroxisome proliferator-activated receptor gamma
PZQ: praziquantel
rAAV: recombinant adeno-associated virus
RG: reference gene
RT-PCR: reverse transcribed polymerase chain reaction
SEA: soluble egg antigens
sncRNA: small non-coding RNAs
TGF- β 1: transforming growth factor β 1
Th: T helper
TLR: Toll-like receptor
TNF- α: tumor necrosis factor- α
UTR: untranslated region.
